# Supplementary material for: Intracellular pocket conformations determine signaling efficacy through the μ opioid receptor
Source: bioRxiv. 2024 Dec 7:2024.04.03.588021. Preprint. [Version 2] doi: 10.1101/2024.04.03.588021 (PMC11642773; doi:10.1101/2024.04.03.588021)
Supplement: Supplement 1 [file NIHPP2024.04.03.588021v2-supplement-1.pdf]

bioRxiv preprint doi: https://doi.org/10.1101/2024.04.03.588021; this version posted December 7, 2024. The copyright holder for this preprint (which was not certified by peer review) is the author/funder, who has granted bioRxiv a license to display the preprint in perpetuity. It is made available under aCC-BY 4.0 International license.

Supporting Information

Figures S1 to S12

Tables S1 to S2

SI References

bioRxiv preprint doi: https://doi.org/10.1101/2024.04.03.588021; this version posted December 7, 2024. The copyright holder for this preprint (which was not certified by peer review) is the author/funder, who has granted bioRxiv a license to display the preprint in perpetuity. It is made available under aCC-BY 4.0 International license.

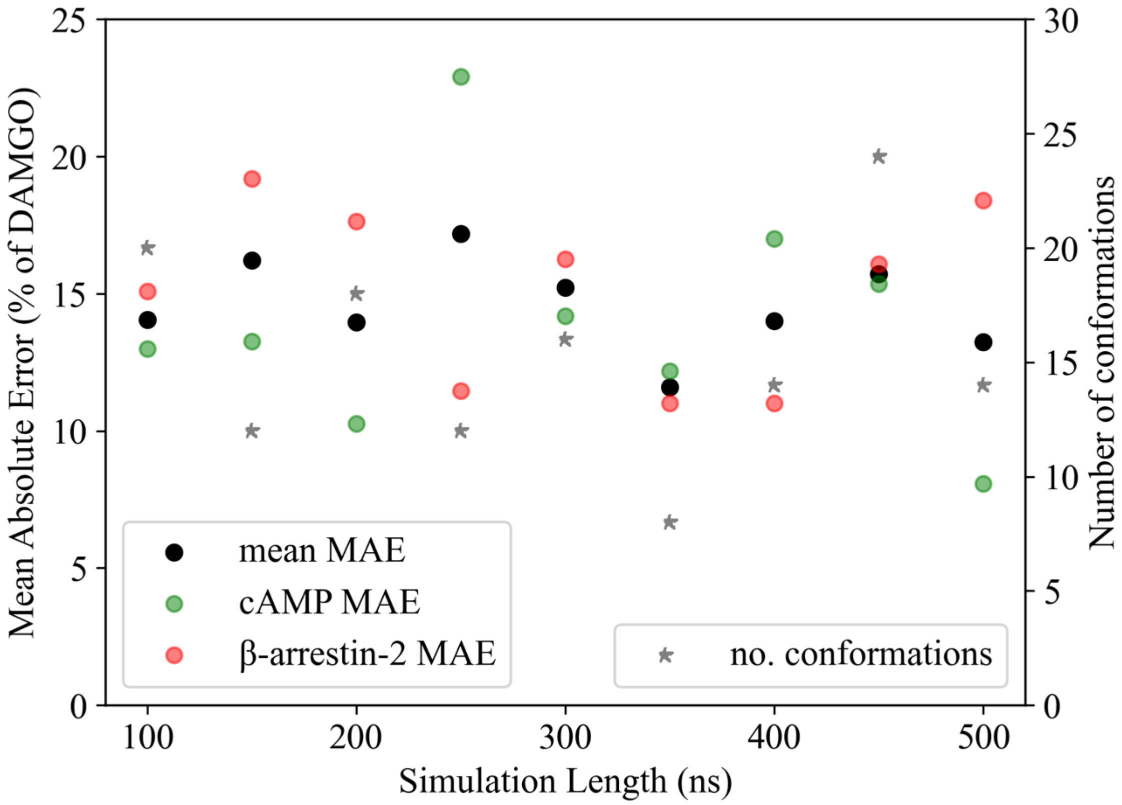

Figure S1 Model parameters and performance as a function of simulation length

bioRxiv preprint doi: https://doi.org/10.1101/2024.04.03.588021; this version posted December 7, 2024. The copyright holder for this preprint (which was not certified by peer review) is the author/funder, who has granted bioRxiv a license to display the preprint in perpetuity. It is made available under aCC-BY 4.0 International license.

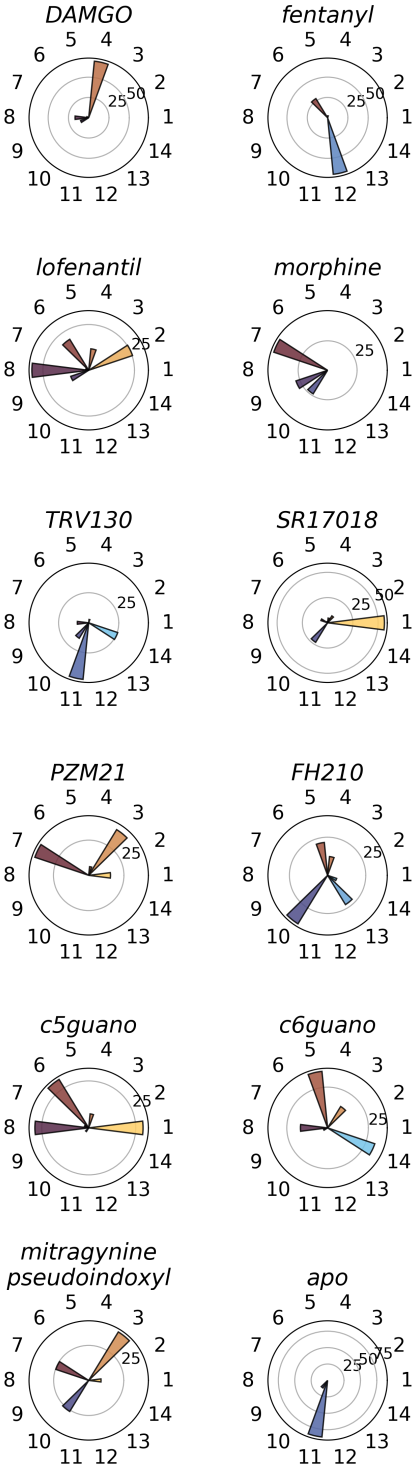

Figure S2 Percentages of simulations of complexes with different ligands in each conformation.

bioRxiv preprint doi: https://doi.org/10.1101/2024.04.03.588021; this version posted December 7, 2024. The copyright holder for this preprint (which was not certified by peer review) is the author/funder, who has granted bioRxiv a license to display the preprint in perpetuity. It is made available under aCC-BY 4.0 International license.

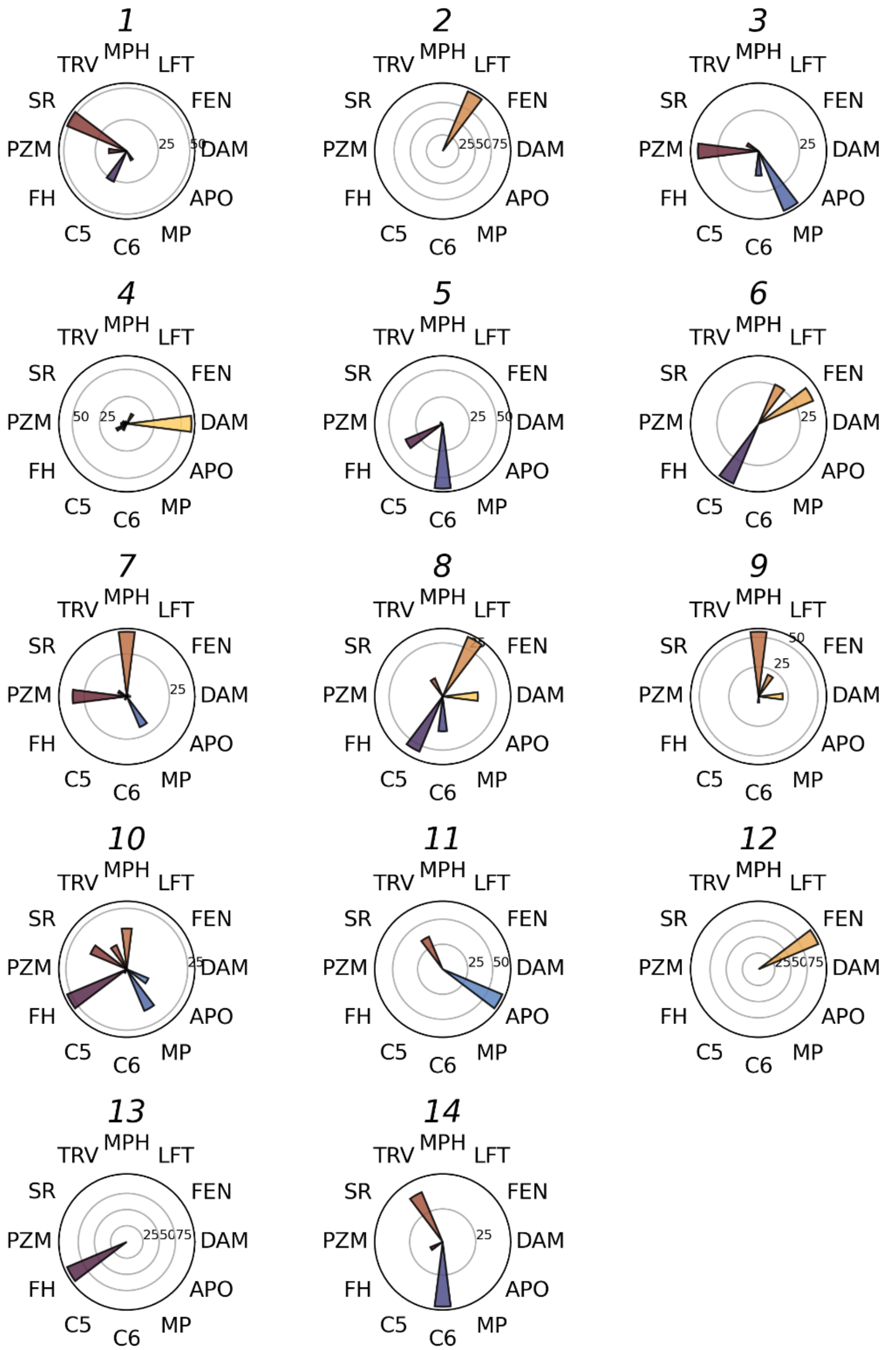

Figure S3 Percentages of conformations accessed in simulations with each complex. Abbreviations: DAM: DAMGO, FEN: fentanyl, LFT: lofentanil, MPH: morphine, TRV: TRV130, SR: SR17018, PZM: PZM21, FH: FH210, C5: c5guano, C6: c6guano, MP: mitragynine pseudoindoxyl, APO: apo.

bioRxiv preprint doi: https://doi.org/10.1101/2024.04.03.588021; this version posted December 7, 2024. The copyright holder for this preprint (which was not certified by peer review) is the author/funder, who has granted bioRxiv a license to display the preprint in perpetuity. It is made available under aCC-BY 4.0 International license.

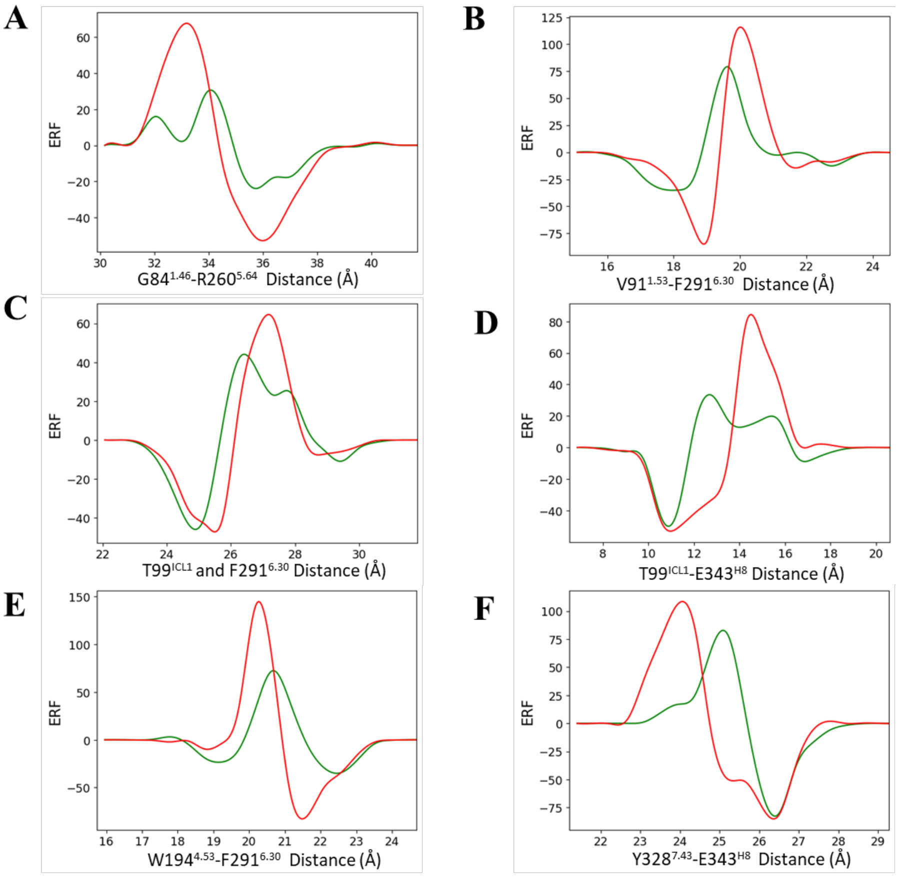

Figure S4 Efficacy response functions for alpha carbon distances with the largest general activation scores: (A) G84$^{1.46}$-R260$^{5.64}$, (B) V91$^{1.53}$-F291$^{6.30}$, (C) T99^{ICL1}-F291$^{6.30}$, (D) T99^{ICL1}-E343^{H8}, (E) W194$^{4.53}$-F291$^{6.30}$, and (F) Y328$^{7.43}$-E343^{H8}. The green and red curves correspond to the G protein and β-arrestin-2 efficacy response functions (ERF), respectively.

bioRxiv preprint doi: https://doi.org/10.1101/2024.04.03.588021; this version posted December 7, 2024. The copyright holder for this preprint (which was not certified by peer review) is the author/funder, who has granted bioRxiv a license to display the preprint in perpetuity. It is made available under aCC-BY 4.0 International license.

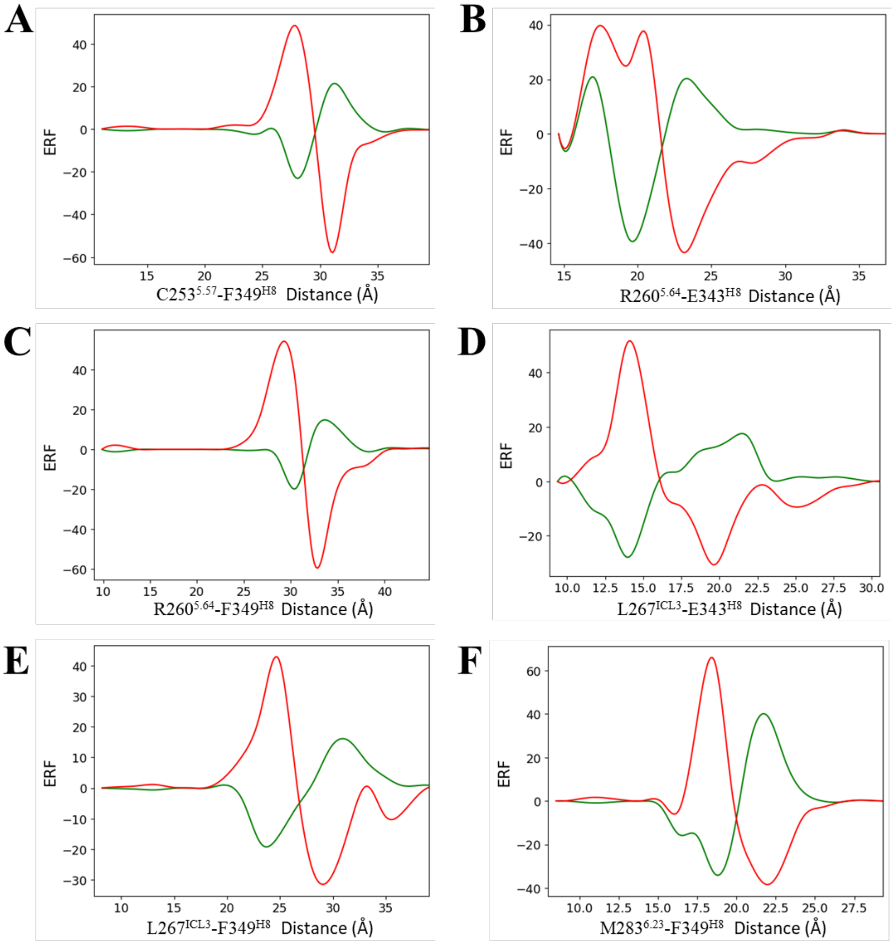

Figure S5 Efficacy response functions for alpha carbon distances with the largest selective activation scores: (A) $C253^{5.57}$-$F349^{H8}$, (B) $R260^{5.64}$-$E343^{H8}$, (C) $R260^{5.64}$-$F349^{H8}$, (D) $L267^{ICL3}$-$E343^{H8}$, (E) $L267^{ICL3}$-$F349^{H8}$, and (F) $M283^{6.23}$-$F349^{H8}$. The green and red curves correspond to the G protein and β-arrestin-2 efficacy response functions (ERF), respectively.

bioRxiv preprint doi: https://doi.org/10.1101/2024.04.03.588021; this version posted December 7, 2024. The copyright holder for this preprint (which was not certified by peer review) is the author/funder, who has granted bioRxiv a license to display the preprint in perpetuity. It is made available under aCC-BY 4.0 International license.

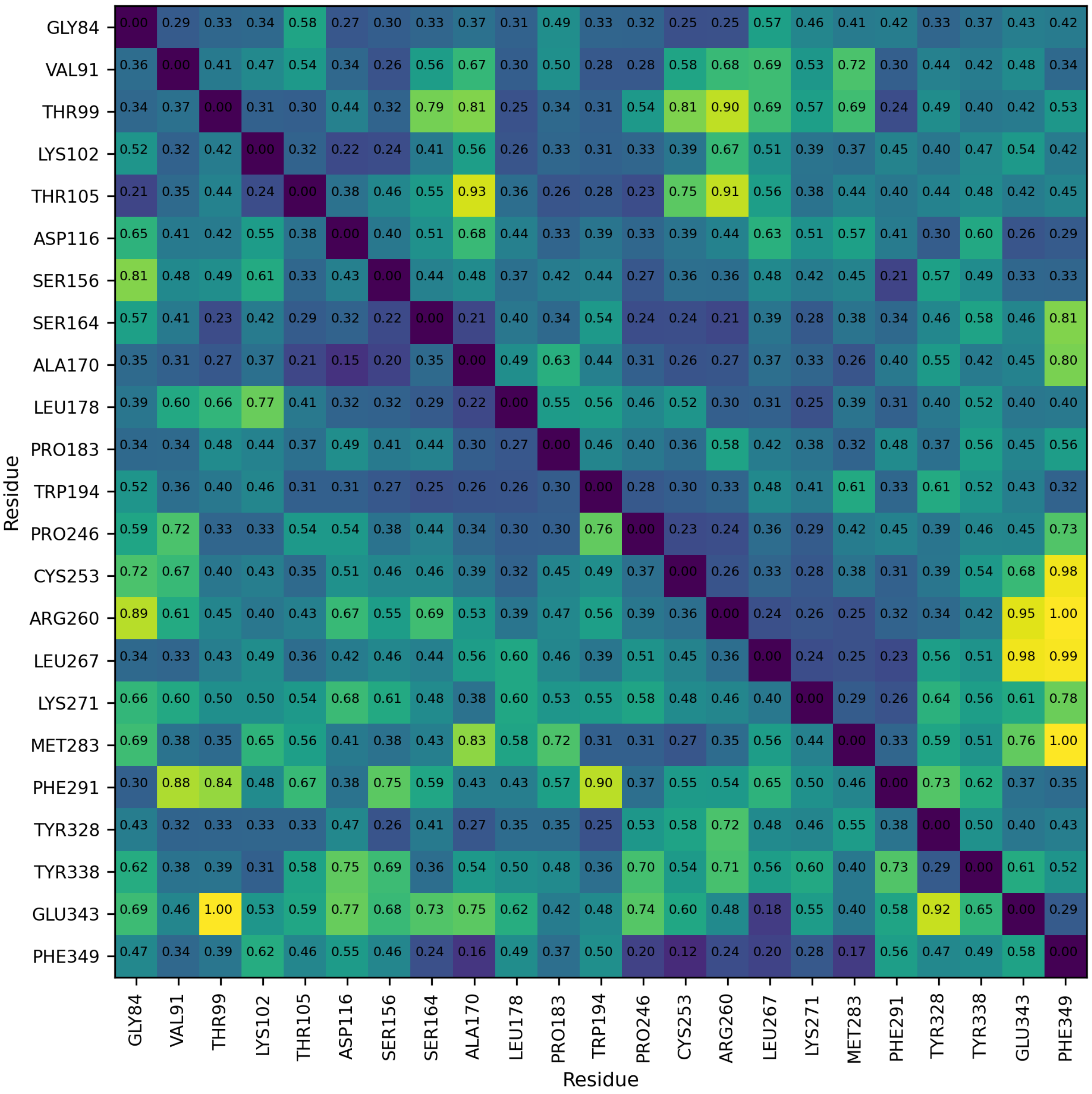

Figure S6 Efficacy response function scores for pairwise distances between alpha carbons from residues in the middle and intracellular end of each helix, with at least one residue in between, and the middle of each intracellular loop. Scores below the diagonal were calculated with the general activation function (Equation 8). Scores above the diagonal were calculated with the selective activation function (Equation 9). Scores from each category were divided by the largest score in the category.

bioRxiv preprint doi: https://doi.org/10.1101/2024.04.03.588021; this version posted December 7, 2024. The copyright holder for this preprint (which was not certified by peer review) is the author/funder, who has granted bioRxiv a license to display the preprint in perpetuity. It is made available under aCC-BY 4.0 International license.

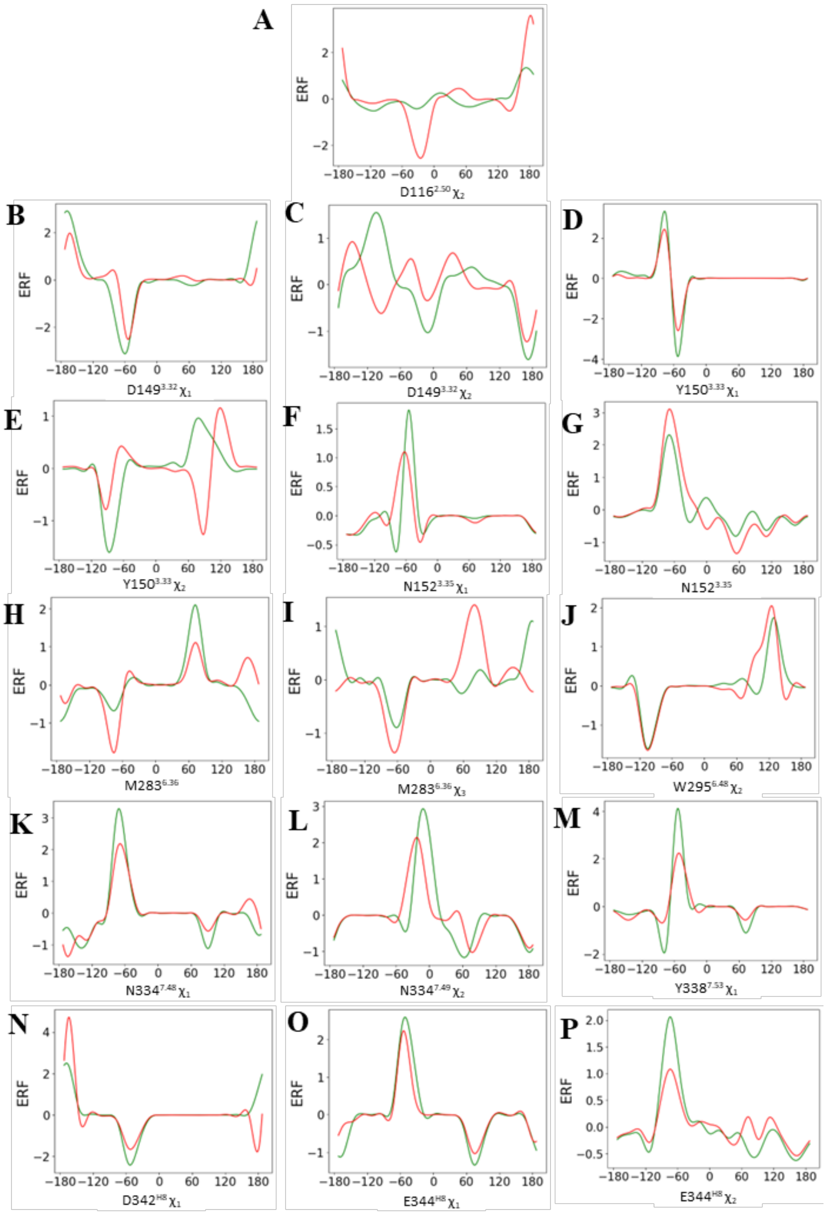

Figure S7 Efficacy response functions for dihedral angles with the largest general activation scores**: (A)** D116$^{2.50}$ χ_2 angle, **(B)** D149$^{3.32}$ χ_1 angle, **(C)** D149$^{3.32}$ χ_2 angle, **(D)** Y150$^{3.33}$ χ_1 angle, **(E)** Y150$^{3.33}$ χ_2 angle, **(F)** N152$^{3.35}$ χ_1 angle, **(G)** N152$^{3.35}$

χ_2 angle, **(H)** M283$^{6.36}$ χ_2 angle, **(I)** M283$^{6.36}$ χ_3 angle, **(J)** W295$^{6.48}$ χ_2 angle, **(K)** N334$^{7.48}$ χ_1 angle, **(L)** N334$^{7.49}$ χ_2 angle, **(M)** Y338$^{7.53}$ χ_1 angle, **(N)** D342^{H8} χ_1 angle**, (O)** E344^{H8}

χ_1 angle, **(P)** E344^{H8} χ_2 angle. The green and red curves correspond to the G protein and β-arrestin-2 efficacy response functions (ERF), respectively.

bioRxiv preprint doi: https://doi.org/10.1101/2024.04.03.588021; this version posted December 7, 2024. The copyright holder for this preprint (which was not certified by peer review) is the author/funder, who has granted bioRxiv a license to display the preprint in perpetuity. It is made available under aCC-BY 4.0 International license.

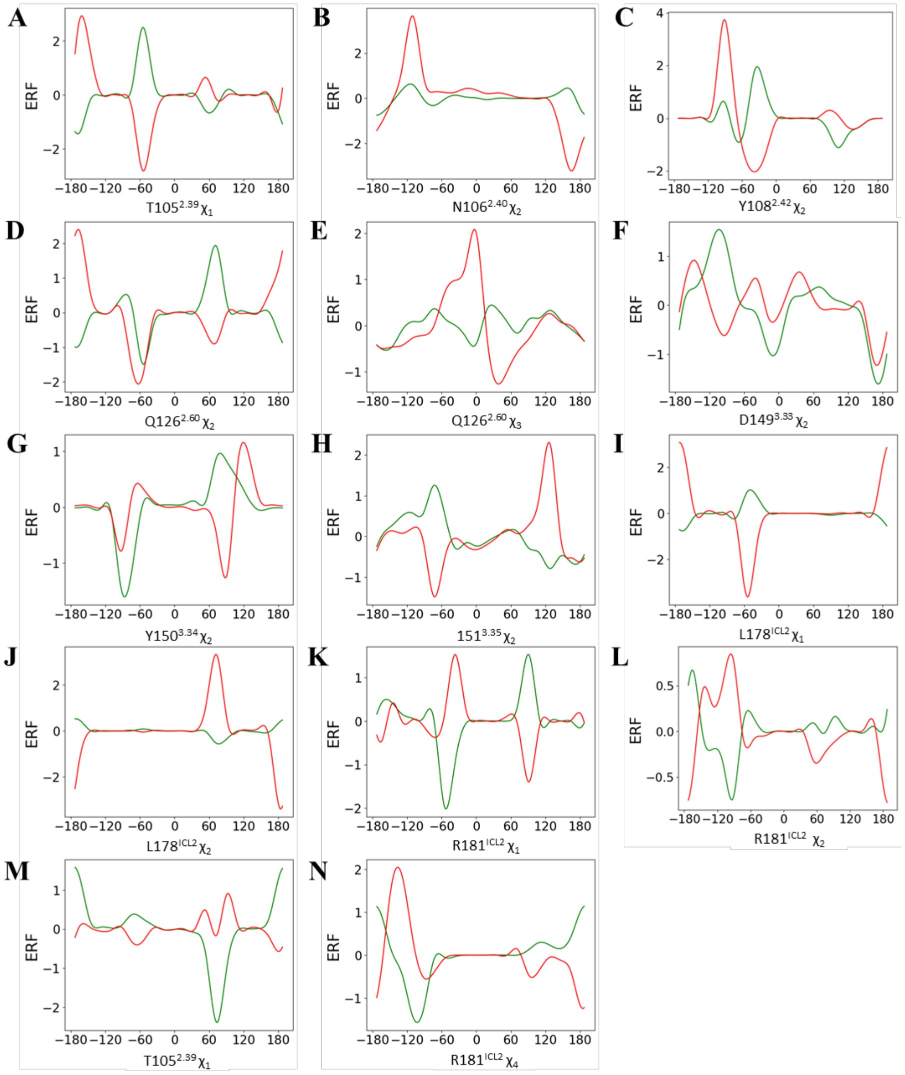

Figure S8 Efficacy response functions for dihedral angles with the largest selective activation scores: (A) T105$^{2.39}$ χ_1 angle, (B) N106$^{2.40}$ χ_2 angle, (C) Y108$^{2.42}$ χ_2 angle, (D) Q126$^{2.60}$ χ_2 angle, (E) Q126$^{2.60}$ χ_3 angle, (F) D149$^{3.32}$ χ_2 angle, (G) Y150$^{3.33}$ χ_2 angle, (H) 151$^{3.35}$ χ_2 angle, (I) L178^{ICL2} χ_1 angle, (J) L178^{ICL2} χ_2 angle, (K) R181^{ICL2} χ_1 angle, (L) R181^{ICL2} χ_2 angle, (M) R181^{ICL2} χ_3 angle, (N) R181^{ICL2} χ_4 angle. The green and red curves correspond to the G protein and β-arrestin-2 efficacy response functions (ERF), respectively.

bioRxiv preprint doi: https://doi.org/10.1101/2024.04.03.588021; this version posted December 7, 2024. The copyright holder for this preprint (which was not certified by peer review) is the author/funder, who has granted bioRxiv a license to display the preprint in perpetuity. It is made available under aCC-BY 4.0 International license.

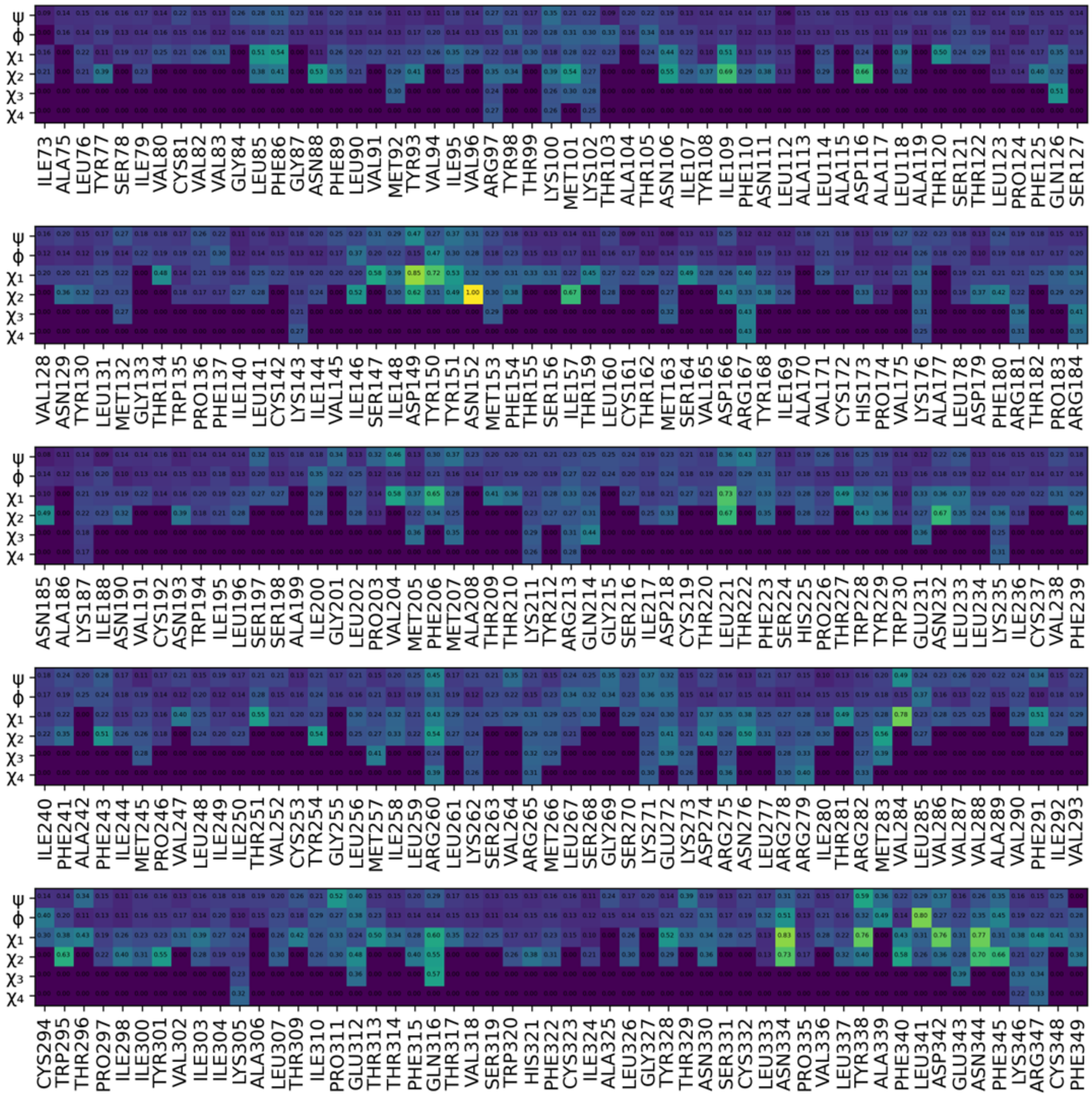

Figure S9 General activation function scores for amino acid dihedral angles.

bioRxiv preprint doi: https://doi.org/10.1101/2024.04.03.588021; this version posted December 7, 2024. The copyright holder for this preprint (which was not certified by peer review) is the author/funder, who has granted bioRxiv a license to display the preprint in perpetuity. It is made available under aCC-BY 4.0 International license.

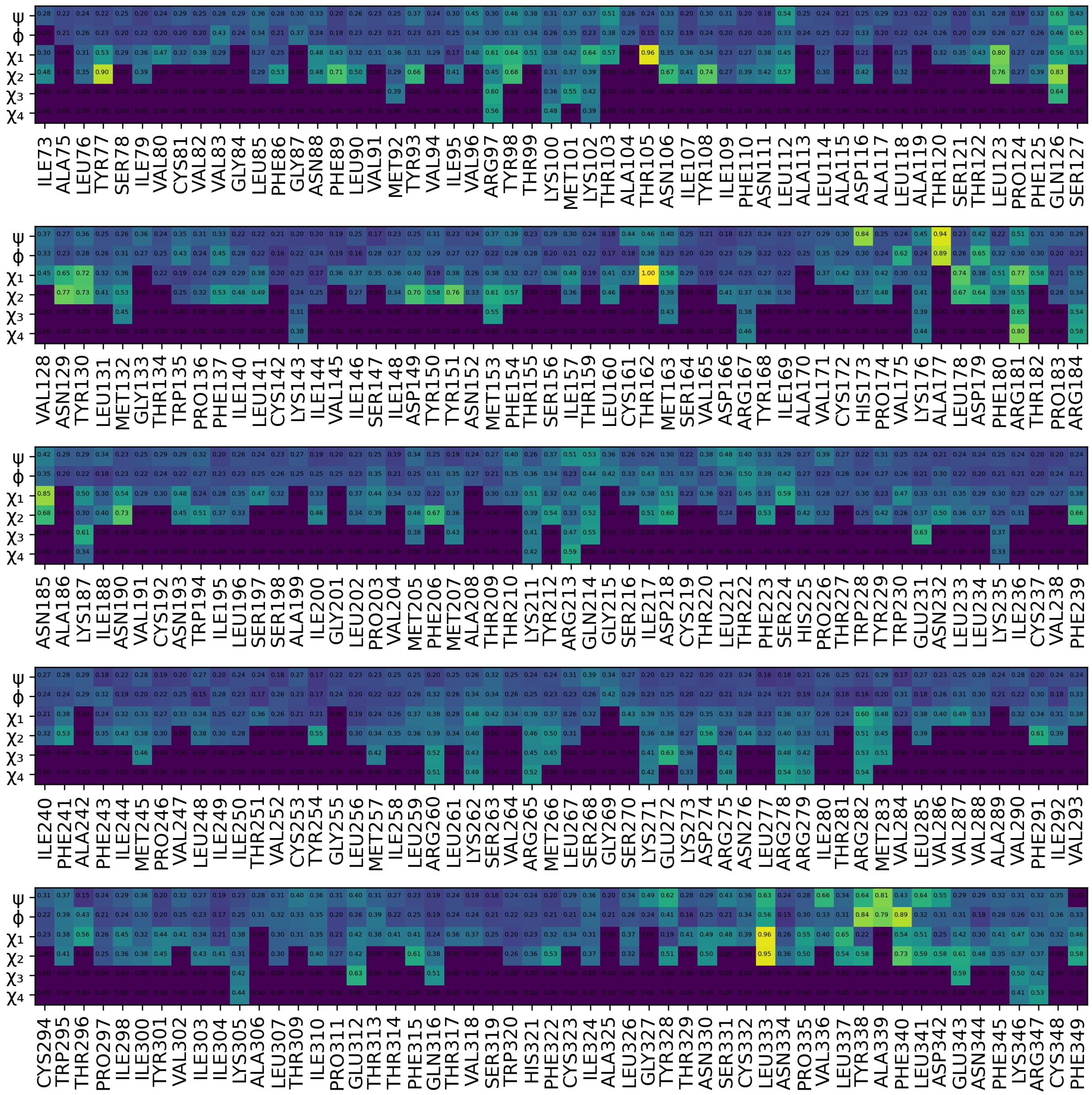

Figure S10 Selective activation function scores for amino acid dihedral angles.

bioRxiv preprint doi: https://doi.org/10.1101/2024.04.03.588021; this version posted December 7, 2024. The copyright holder for this preprint (which was not certified by peer review) is the author/funder, who has granted bioRxiv a license to display the preprint in perpetuity. It is made available under aCC-BY 4.0 International license.

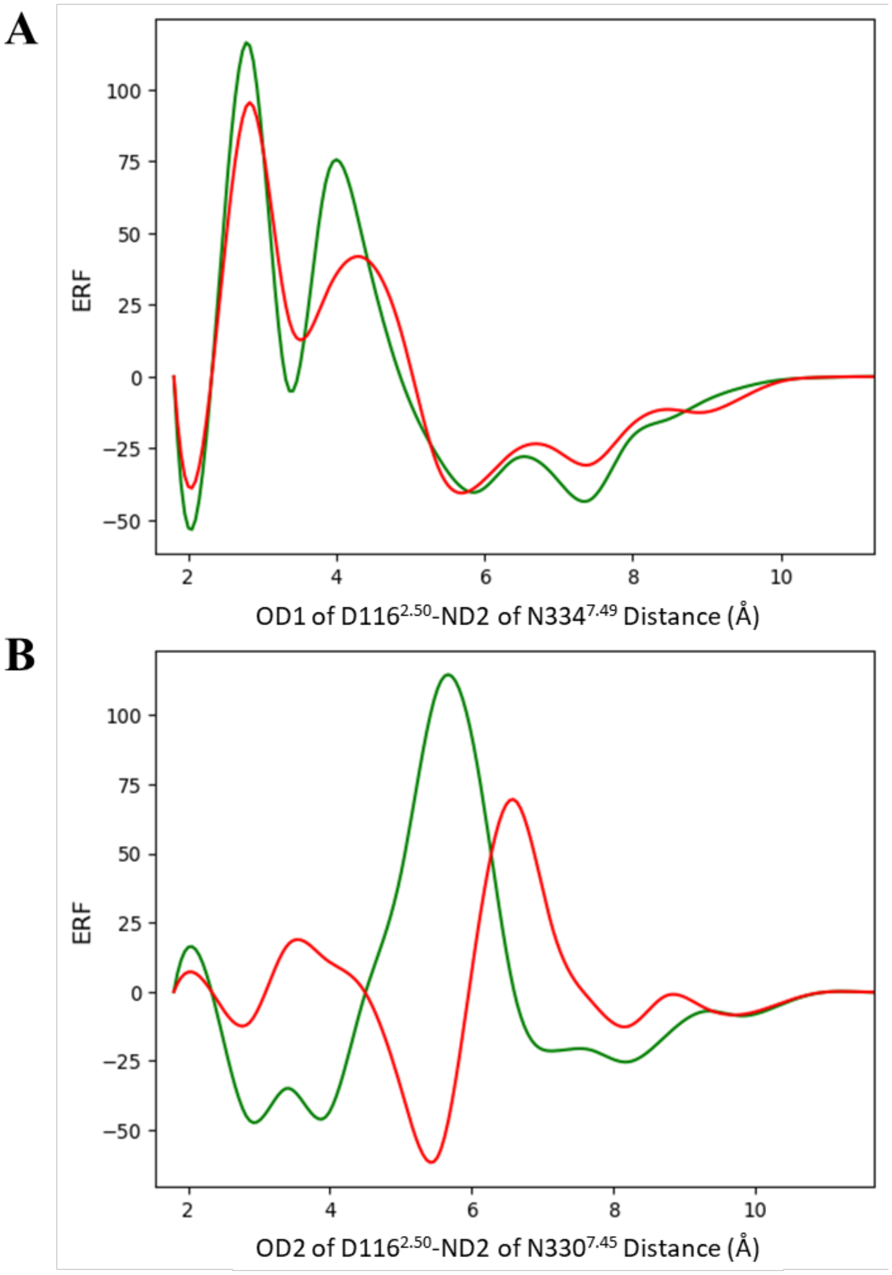

Figure S11 Efficacy response functions of the distance between sodium pocket polar atoms**:** (**A)** OD1 of D116$^{2.50}$ and ND2 of N334$^{7.49}$ and (**B)** OD2 of D116$^{2.50}$ and ND2 of N330$^{7.45}$. The green and red curves correspond to the G protein and β-arrestin-2 efficacy response functions (ERF), respectively.

bioRxiv preprint doi: https://doi.org/10.1101/2024.04.03.588021; this version posted December 7, 2024. The copyright holder for this preprint (which was not certified by peer review) is the author/funder, who has granted bioRxiv a license to display the preprint in perpetuity. It is made available under aCC-BY 4.0 International license.

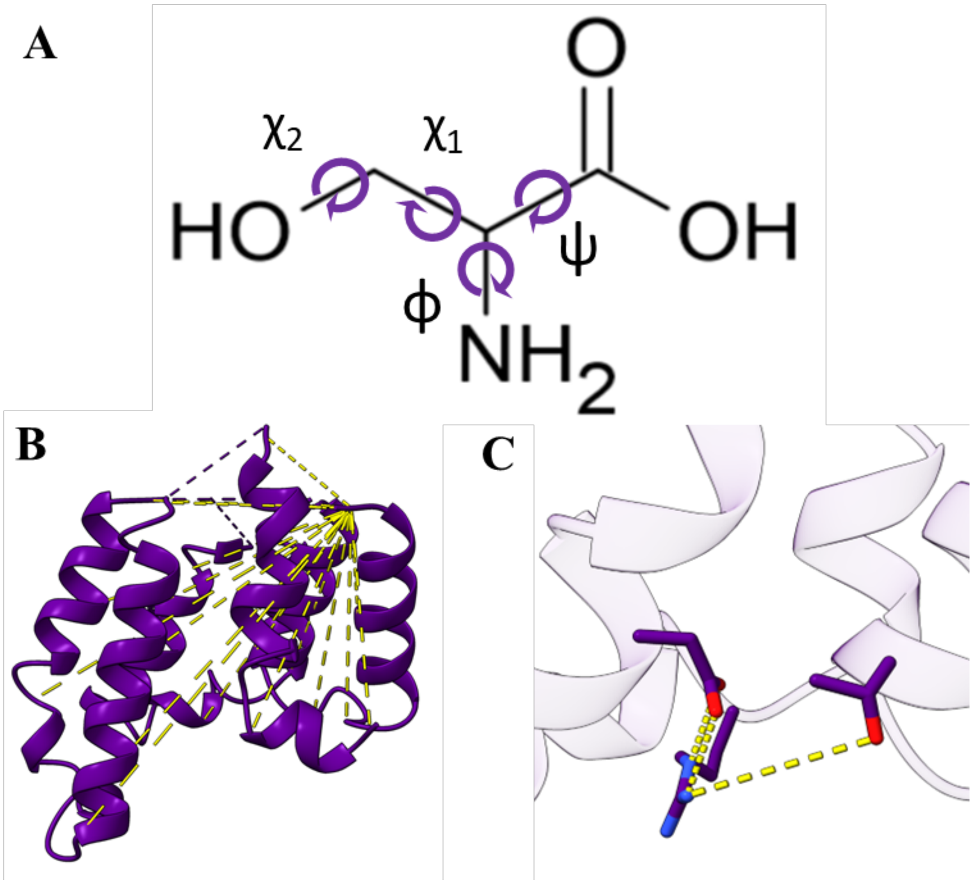

Figure S12 Representations of features used to discretize configurations into conformations: **A)** dihedral angles, **B)** alpha carbon distances, and **C)** distances between polar atoms in the intracellular region of the μOR.

bioRxiv preprint doi: https://doi.org/10.1101/2024.04.03.588021; this version posted December 7, 2024. The copyright holder for this preprint (which was not certified by peer review) is the author/funder, who has granted bioRxiv a license to display the preprint in perpetuity. It is made available under aCC-BY 4.0 International license.

Ligand	Structure	PDBID	G protein E_{max}	β-arr-2 E_{max}	Sources
FH210	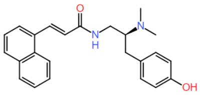	7scg	80 (4)*	18 (2)*	[39]
mitragynine pseudoindoxyl	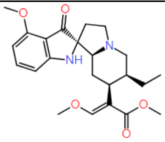	7t2g	81 (4)	0 (0)**	[67]
lofentanil	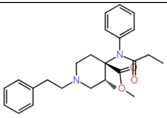	7t2h	115 (2)***	110 (3)	[27]
c6guano	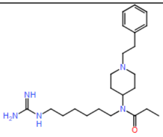	7u2k	137 (2)	19 (1)	[28]
c5guano	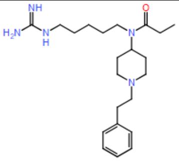	7u2l	104 (3)	54 (3)	[28]
fentanyl	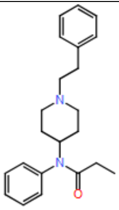	8ef5	92 (4)	60 (2)	[26,28,68–70]
morphine	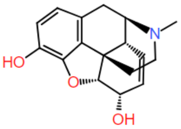	8ef6	97 (4)	28 (3)	[26,59,68,70,71]
TRV130	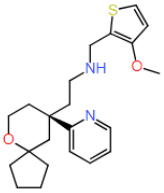	8efb	86 (8)	0 (0)**	[26,59,70,72]
SR10718	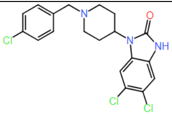	8efl	62 (12)	12 (1)	[26,68,70]

bioRxiv preprint doi: https://doi.org/10.1101/2024.04.03.588021; this version posted December 7, 2024. The copyright holder for this preprint (which was not certified by peer review) is the author/funder, who has granted bioRxiv a license to display the preprint in perpetuity. It is made available under aCC-BY 4.0 International license.

PZM21	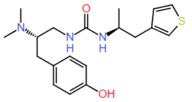	8efo	91 (7)	0 (0)**	26,59,70,71
DAMGO	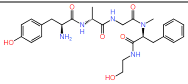	8efq	100	100	

** Converted to DAMGO reference from median experimental reference*

*** Not detectable*

**** Average across multiple Gα subtypes*

Table S1: Summary of structures and efficacy data used for MDS and model training. Emax reported as median Emax (median STD)

Ligand	G protein Recruitment (% of DAMGO)	Uncertainty	Assay	β-arrestin-2 recruitment (% of DAMGO)	Uncertainty	Assay	Source
MP	81	4	cAMP			NR	67
TRV130	66	2	cAMP	ND	ND	BRET	59
	86	3	cAMP	ND	ND	PathHunter	72
	86	5	cAMP	16	3	FRET	70
	86	6	cAMP	ND	ND	NanoBit	26
PZM21	76	3	cAMP	ND	ND	BRET	59
	84	7	cAMP	14	3	BRET	70
	97	NR	cAMP	ND	ND	NanoBit	71
	112	8	cAMP	ND	ND	NanoBit	26
SR17018	105	3	cAMP	10	6	PathHunter	68
	62	12	cAMP	12	1	BRET	70
	49	12	cAMP	13	5	NanoBit	26
Morphine	100	NR	cAMP	14	NR	NanoBit	71
	97	1	cAMP	24	1	PathHunter	68
	97	4	cAMP	28	3	BRET	70
	107	4	cAMP	29	1	NanoBit	26
	89	2	cAMP	39	6	BRET	59
Fentanyl	97	5	cAMP	60	2	PathHunter	68
	NR	NR	NR	59	NR	NanoBit	69
	87	4	cAMP	43	4	NanoBit	26

bioRxiv preprint doi: https://doi.org/10.1101/2024.04.03.588021; this version posted December 7, 2024. The copyright holder for this preprint (which was not certified by peer review) is the author/funder, who has granted bioRxiv a license to display the preprint in perpetuity. It is made available under aCC-BY 4.0 International license.

	86	2	cAMP	119	3	Tango	28
	100	5	cAMP	70	4	NanoBit	70
LFT	115	2	BRET	110	3	BRET	27
c5guano	104	3	cAMP	54	3	Tango	28
c6guano	137	2	cAMP	19	1	Tango	28
FH210	80*	4	IP-One	18*	2	PathHunter	39

NR Not reported

ND Not detectable

* Converted to DAMGO reference from median experimental reference

Table S2: Experimental cAMP and βarr2 efficacies used to compute the median experimental efficacies for model training.
